# The Effect of Positive Working Conditions on Work Engagement and Teaching Classroom Practices: A Large Cross-Sectional Study in Switzerland

**DOI:** 10.3389/fpsyg.2019.02129

**Published:** 2019-09-20

**Authors:** Loredana Addimando

**Affiliations:** Center for Innovation and Research in Education, Department of Teaching and Learning, University of Applied Science and Arts of Southern Switzerland, Locarno, Switzerland

**Keywords:** teaching classroom practices, teacher job satisfaction, social support, work engagement, self-determination theory

## Abstract

**Introduction:**

Teaching methods and techniques represent important classroom practices that promote both academic achievement and the efficacy of learning processes: the use of a complex array of teaching practices by educators in the classrooms has been frequently associated with better student academic outcomes (Hattie, 2012, 2009). So far, less is known about the psychological aspects linked to the working endeavors able to promote the adoption of different teaching techniques in educational contexts.

**Aim:**

The present cross-sectional quantitative research aimed at estimating the cumulative network of associations between psychological resources at work (e.g., social support, sense of autonomy, and job satisfaction) and both work engagement and the use of teaching classroom practices.

**Sample and Method:**

The sample was composed of in-service teachers (*N* = 1,370) recruited in primary and lower secondary public schools in urban and suburban areas of Canton Ticino (Switzerland). Self-administered quantitative measures were used to evaluate the following constructs: social support, job satisfaction, teachers’ autonomy, work engagement, and teaching classroom practices. The data were analyzed using structural equation modeling with decomposition analysis of total effects in direct and indirect effects.

**Results:**

The hypothesized structural model reported a good fit with the empirical data (normed χ^2^ (NC) = 2.7; root mean square error of approximation = 0.051, normed fit index (NNFI) = 0.951, non normed fit index = 0.950, comparative fit index = 0.968) and support the interpretation of the structural model. The main results revealed a large positive association between psychological resources at work and teaching classroom practices (β = 0.37). The level of work engagement seemed to be a minor element in promoting the use of teaching classroom practices (β = 0.10).

**Discussion:**

Overall, the results provide a fresh piece of evidence for the association between working conditions and teaching practices. In fact, teachers who perceive a supportive and satisfying working environment (both in terms of internal and external resources) are more likely to be engaged in their activities, and this in turn leads to a more heterogeneous array of teaching practices with students. The practical implications in terms of professional training and on-field intervention are discussed.

## Introduction

The teaching and learning processes in the school setting are a typical object of study in many disciplines of the human and social sciences. Psychology, sociology, and pedagogy agree that teacher resources, competencies, skills, and behavioral repertories, along with students’ needs, are considered critical points of paramount importance for research in the education field. Being an effective teacher today requires the implementation of different teaching strategies and techniques (e.g., classic lecture, case-study, simulation, and discussion) to achieve academic goals and support students’ learning (Wray et al., 2000). From a formative point of view, teachers’ teaching skills are conceptualized as the ability to implement different practices depending on the specific needs of the students (Stronge, 2018). Coherently with the idea of studying factors affecting the experience of being a professional teacher, the scientific literature has mainly focused on understanding their professional characteristics as being indicative of their conceptions and professional competencies (Park and Oliver, 2008; Fiorilli, 2009). Other research has been conducted on linking practices to skills learned by students as an indication of students’ progress (e.g., OECD programs for international student assessment, such as the PISA project). That said, fewer studies have focused on exploring the psychological, contextual, and organizational features of teachers’ work that might promote or inhibit the adoption of a variety of teaching practices and techniques. Although the scientific literature extensively examines the connection between teacher practices and classroom characteristics (Allen, 2010; Dudek et al., 2019), teacher beliefs (Farrell and Guz, 2019; Mills et al., 2019), or the presence of technological devices (Uerz et al., 2018; Nelson et al., 2019), much remains to be learned about psychological resources that might promote or inhibit the use of practices in classroom teaching. Studying how teaching strategies are linked to psychological and environmental resources is critical to determining which strategies to favor over others and expand the implementation of flexible teaching practices in empirical contexts.

According to the paradigms of positive psychology and by adopting the theoretical framework of self-determination theory (SDT) (Ryan and Deci, 2000), the present research aims at exploring the patterns of association among the variety of practices adopted in the classroom, psychological resources related to contextual working conditions (i.e., social support, sense of autonomy, job satisfaction), and levels of work engagement. This is the focus because a satisfied and engaged person not only performs better in teaching (Bakker et al., 2008) but also have a subsequent impact on students’ learning (Mercer, 2010). The expected results are aimed at identifying a model capable of evaluating psychological resources in relation to the use of a wide array of teaching practices in the classroom.

### Literature Review

The study of teaching practices is the cornerstone and antecedent of studies on the evaluation of the effectiveness of teaching and learning processes. Longitudinal research at the international level has reported that improvement in education and the academic success of students is closely related to the daily processes that take place in the classroom (Willis, 2017; Zierer and Wisniewski, 2019). In other words, a connection between the daily training practice and the scholastic and learning results of the students has steadily emerged from the field.

In recent years, Hattie (2012, 2008) and later Masters et al. (2015) conducted over 800 meta-analyses on a set of 52,637 international scientific studies (i.e., mainly in the Anglo-Saxon context) to identify the aspects that, more so than others, have a positive impact on learning. Specifically, Masters et al. (2015) studied the teacher–student relationship, identifying the following 12 elements (hierarchically ordered on the basis of their impact-effect size) able to influence learning in a direct way: first, the expectations of the students (+1.44), followed by class discussions (+0.80), the clarity of the teacher (+0.75), the ability to give feedback (+0.75), skills (+0.62), classroom management (+0.52), cooperative learning (+0.40), homework (+0.29), exercise/rest ratio (+0.28), group skills (+0.12), rejection (−0.13), and mobility (school change, −0.34). In addition, the study identified the following five emerging characteristics of “excellent teachers” (see Masters et al., 2015): the ability to identify the essential representations of their students; the ability to guide learning through the interactions that take place in the classroom; the ability to provide continual feedback, and the ability to demonstrate empathy and express positive emotions toward students and families; and finally, the ability to influence students’ results and to know how to “make a difference.” All these aspects can be considered complex constructs resulting from interactions among cognitive aspects, emotions, empirical experiences, and attitudes of teachers.

Another interesting line of research has focused on the relationship between students and their teachers as a core critical determinant in the development of teachers’ behavior and performance (Douglas et al., 2008; Pianta et al., 2008). In this sense, the goodness of fit theory (Thomas and Chess, 1986; Lerner and Lerner, 2018) has long described how the teaching/learning process is, to a large extent, influenced by the student–teacher degree of fit and relationship. The participation of the students and the flexibility of the training devices can be the focus of both educational objectives (Saravani and Haddow, 2011) and interactive orientation to competence, which refers to the way in which teachers and students work together on ideas and knowledge or mis-knowledge (Molinari et al., 2013). In particular, innovative teaching practices must be studied in their developmental contexts (Honigsfeld and Dove, 2019) since adopting innovations in teaching depends on the sustainability of contextual conditions as well as the attitude of the teachers, which is also the result of their current teaching experience (Ghavifekr and Rosdy, 2015). Teaching practices are therefore also influenced by a teacher’s expectations, and the concepts become powerful mediators of the teacher–student relationship (Roorda et al., 2011). In fact, the extent to which teachers feel free to use new and innovative teaching practices is determined not only by their professional skills and knowledge but also by other psychological drivers (e.g., motivation and feelings of efficacy; Pyhältö et al., 2012).

#### Self-Determination Theory: Social Support, Job Satisfaction, Teachers’ Autonomy, and Work Engagement as Shaping Elements of Teacher Practices

In recent years, positive psychology (i.e., the scientific study of human resources and optimal functioning; Seligman and Csikszentmihalyi, 2014) has gained increased attention in educational contexts. This approach has tried to counterbalance the traditional focus of psychology on disease, damage, or disorder by paying particular attention to the functional facets of the human experience. These recent shifts have also been adopted by applied psychology to “study positively oriented human resource strengths and psychological capacities that can be measured, developed, and effectively managed for performance improvement in today’s workplace” (Luthans, 2002, p. 698). One important contribution that emerged from this new conceptual wave was SDT (Ryan and Deci, 2000) as a general functioning-focused framework for the comprehension of people’s behavioral tendencies and innate psychological needs. In addition, the SDT “is concerned primarily with explicating the psychological processes that promote optimal functioning and health” (Deci and Ryan, 2002, p. 262). In other words, SDT represented a general approach to the comprehension of human behaviors that was based on organismic metatheory and that emphasized the centrality of evolved inner psychological resources for individual development in relation to behavioral self-regulation (Ryan et al., 1997). The core idea of STD is that human beings “are assumed to be active, growth-oriented organisms who are naturally inclined toward the development of an organized coherence among the elements of their psychological makeup and between themselves and the social world” (Deci and Ryan, 2002, p. 262). From this perspective, psychological needs represent the basic elements for self-motivation and goal-oriented behaviors in a given environment and provide a possible explanation for context-specific behavioral decisions. Some examples of empirical application of SDT in educational settings include studies on student motivation (Reeve et al., 2018), teachers’ best practices (Stupnisky et al., 2018) and innovative behaviors (Klaeijsen et al., 2018), leadership (Eyal and Roth, 2011), classroom processes (Pianta and Hamre, 2009) and teaching style (Bartholomew et al., 2018), and student violent behaviors (Assor et al., 2018). According to SDT, human beings can experience different types of motivation with respect to their work. The presence of the different types of motivation is important given that, compared to controlled regulation (introjected and extrinsic motivation), autonomous regulation (intrinsic and identified motivation) leads to more positive individual and organizational outcomes (Slemp et al., 2018). From this point of view, teachers’ experiences seem to be deeply affected by perceived working conditions and the presence of specific psychological resources. In addition, SDT provided an integrated theoretical framework able to conceptualize what stances stand behind a behavior and how people make sense of their own and others’ behavior (Deci et al., 1989). As a result of such important conceptual innovation, a full spectrum of new constructs emerged, supporting both the comprehension of the educational field and teachers’ behavior in the workplaces.

More specifically, SDT identified three general psychological domains able to influence external behaviors: need for competence (Elliot et al., 2002), social relatedness (Baumeister and Leary, 1995), and autonomy (Deci and Ryan, 1987). The need for autonomy refers to the need to have a sense that one can exercise free will and that any activity one undertakes is freely chosen rather than imposed. The need for relatedness is the need to feel a sense of belonging, simply to be loved and cared for (Van den Broeck et al., 2010). Finally, the need for competence is the need to develop mastery over tasks that are important to one (Deci and Ryan, 2002). These areas were saturated by a constellation of psychological constructs that appear to be of paramount importance for “facilitating optimal functioning of the natural propensities for growth and integration, as well as for constructive social development and personal well-being” (Ryan and Deci, 2000, p. 68). The present study selected two of three domains (i.e., social relatedness and perceived autonomy) presented by the SDT as specific areas linked to working conditions in the educational setting. In fact, the third domain (e.g., need for competence) seems to be more focused on internal aspects of individuals and less related to mechanisms shaping professional teaching practices. From this point of view, other constructs (e.g., job satisfaction, social support, and work engagement) appear more informative in relation to teacher work and the use of different practices. In fact, teaching work is entirely based on relationships, given that teachers engage in continuous interaction with students, families, and colleagues. For instance, Klaeijsen et al. (2018) report that job satisfaction affects both intrinsic motivation and occupational self-efficacy and that the latter strongly supports innovative behavior in classrooms. In this sense, Pindek et al. (2019) affirm that: “It is certainly not surprising that inadequate resources would adversely affect job performance and that […] perhaps the most important contribution is to show that the most common harmful elements in terms of motivation do arise from colleagues and managers” (p. 91).

The term job satisfaction usually refers to the degree to which employees like the components of their job (Spector, 1997). Over time and across different paradigms, many definitions of the construct have also been adopted (Van Saane et al., 2003). Even with the variations in this broad range of available definitions, the majority of them share the knowledge that job satisfaction is essentially an effective and positive job-related reaction to the workplace (Addimando, 2013; Judge et al., 2017) that explains how people feel about their work (Kianto et al., 2016). Job satisfaction in teaching is a crucial focus for educational research, mainly because of the benefits, for both teachers and students, that “satisfied” teachers are known to contribute to organizational outcomes (Zee and Koomen, 2016).

Other important constructs were represented by both the internal support provided by professional figures such as peers, senior teachers, tutors, head teachers, or psychologists and the external support provided by private relational networks such as partners, relatives, and friends. As confirmed by Betoret (2006), the feeling of being exhausted or oppressed by the demands of the job is markedly lower when a social support network of colleagues, superiors, and tutors is available (Fiorilli et al., 2015). Social support is also associated with the sense of being related to others (Wentzel, 1998) and appears to be an important promoting factor of well-being and efficacy in schools (Collie et al., 2012).

Similarly, job autonomy was found to be significantly and positively correlated with organizational commitment and work engagement (Gillet et al., 2018; Van Wingerden et al., 2018). Teachers’ job autonomy is the freedom given to teachers to make their own decisions while doing their job, whereby they elect their practices, design their tasks and/or materials, evaluate outcomes, cooperate with others to solve problems, take responsibilities for their own decisions (Balkar, 2015), get involved in organizational decision making (Tims et al., 2015), and improve their professional skills (Guglielmi et al., 2016). When autonomy is enhanced, teachers are more involved in achieving new skills, and they are more responsible for difficulties at work (Swartz and Perkins, 2016). Job autonomy has been consistently linked to employee satisfaction as a positive factor in job satisfaction (Dou et al., 2017).

Finally, throughout the decades, organizational studies have found that work engagement is defined as a positive, fulfilling, work-related state of mind that is characterized by vigor, dedication, and absorption (Lisbona et al., 2018; Carmona-Halty et al., 2019). Rather than a transitory and specific state, engagement refers to a more persistent and pervasive affective-cognitive status that is not specifically focused on any object, event, individual, or behavior. Work engagement was operationalized using three different work-related domains: vigor, dedication, and absorption. Vigor is characterized by high levels of energy and mental resilience while working, the willingness to invest effort in one’s work, and persistence even in the face of difficulties. Dedication refers to being actively involved in one’s work and experiencing a sense of significance, enthusiasm, inspiration, pride, and challenge. Finally, absorption is characterized by being fully concentrated and happily engrossed in one’s work, whereby time passes quickly, and one has difficulties with detaching oneself from work. Accordingly, vigor and dedication are considered direct opposites of the core burnout dimensions of exhaustion and cynicism, respectively (Maslach et al., 2001). The scientific literature has demonstrated the effect of job satisfaction and teachers’ autonomy on work engagement (Alonderiene and Majauskaite, 2016; Van den Broeck et al., 2016; Cassidy et al., 2017; Vangrieken et al., 2017) and the protective role of satisfaction in relation to teacher burnout (Fiorilli et al., 2015, 2017a).

The intrinsic features of teachers’ job autonomy, satisfaction, and social support (as framed within the SDT) as antecedents of work engagement support the idea that such constructs could be conceived as important structural antecedents of teachers’ behaviors, especially in relation to their roles in favoring positive organizational outcomes and student achievement. In addition, teaching strategies and techniques are highly influenced by the personal evaluation of psychological inner states (Stronge, 2018), and there is a lack of empirical studies focusing on how teachers’ appraisals of their psychological resources linked to working environments (expressed in terms of autonomy, satisfaction, and social support) affect the engagement and teaching practices adopted by the teachers. Hence, it is relevant to study whether and to what extent psychological intrinsic inner states are related to engagement and teaching processes in a fully comprehensive model tested on empirical data gathered in real educational settings.

### Research Objectives and Hypotheses

In line with the aforementioned reviewed literature, the present study aimed to focus on the following three objectives: (1) to examine the relationship between psychological intrinsic basic factors (perceived job autonomy, satisfaction, and social support) and work engagement; (2) to examine the relationship between work engagement and teaching practices; and (3) to examine an integrated cumulative model assessing the total, direct, and indirect effects on the appraisal of “psychological resources” linked to the working environment, engagement, and teachers’ direct practices in the classroom. Following that, three directional hypotheses were formulated:

H1: Perceived job autonomy, social support, and job satisfaction are directly and positively associated with work engagement.
H2: Work engagement is positively associated with the heterogeneity of educational practices adopted by teachers in the classroom.
H3: Psychological resources linked to working environment and engagement favor the use of different didactic practices in the classroom.

**FIGURE 1:**
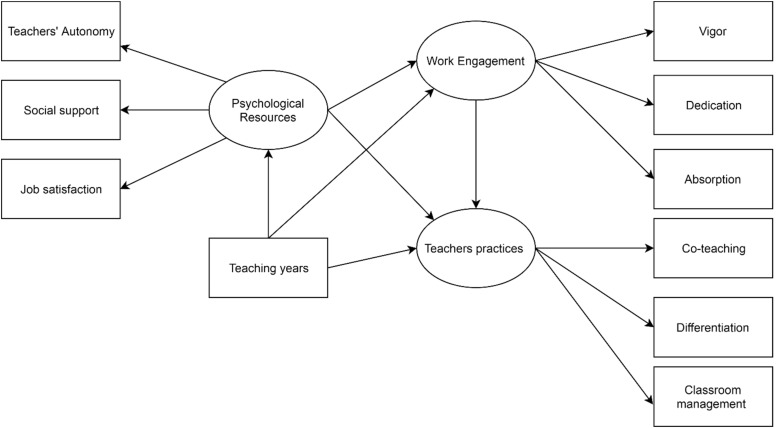
Hypothesized conceptual model of association among considered variables.

The full conceptual model (Figure 1) was designed with three latent endogenous variables and their corresponding empirical indicators. In line with social determination theory, the first latent variable was the appraisal of “psychological resources” linked to working environments, as estimated by the following three empirical measures: teachers’ perceived job autonomy, job satisfaction, and social support. Work engagement was estimated using the standard model of measurement (i.e., physical, cognitive, and emotional energy) expressed by teachers in performing their jobs (Lisbona et al., 2018). The last latent target variable (criterion) of the study included teaching practice techniques and strategies. The variable years of teaching were modeled as a potential source of covariation. This procedure allowed us to analyze segregated data, given that it is a more informative technique (Pepe and Addimando, 2014; Pearl et al., 2016; Fiorilli et al., 2017b; Pepe et al., 2018) and a means of controlling for the Yule–Simpson effect (i.e., a statistical association that holds for the full sample but is reversed in all subpopulations, Simpson, 1951).

## Materials and Methods

### Participants and Procedures

Participants were in-service teachers from mandatory public schools in Canton Ticino, Switzerland. The number of teachers enrolled in the study was 1,370 (37.7% of the whole population). The respondents selected in this study were teachers in preprimary (18%), primary (43%), and lower secondary schools (39%). The sample was composed of 25% of men (*n* = 342) and 75% of women (*n* = 1,028), with an average age of 42.37 (ds = 10.59) years and a seniority (mean of teaching years) of 15.19 (ds = 10.72) years. The study conducted was a quantitative, cross-sectional study, meaning that data from the participants were collected at one specific point of time. All self-reported questionnaires were administered on-site during school hours. Two trained researchers distributed the self-reported measures to the participants in approximately 50 different school settings. The mean time for completing the research protocol was close to 45 min. Inclusion criteria for involvement in the study were: (1) being an in-service teacher, (2) having worked for at least 1 year, and (3) being in charge of a primary or lower secondary classroom.

### Quantitative Measures

#### Inventory of Teaching Practices

Teaching practices were evaluated with different tools focused on traditional teaching practices (e.g., instructional teaching, one-way lessons, and traditional class), collaborative practices (e.g., co-teaching and team teaching, and projects), and innovative teaching (e.g., ICT in teaching, differentiation, and personalized learning). Due to the unavailability of quantitative tools to observe practices in Ticino, the study used the CWSEI Teaching Practices Inventory (Wieman and Gilbert, 2014), a tool specifically designed to evaluate teaching practices used in undergraduate science and mathematics courses. Since practices refer to observable behaviors, and we did not have a set of practices defined beforehand, we preferred to integrate the CWSEI with other tools. In particular, teachers’ strategies and activities were evaluated with items taken from a validated Italian questionnaire specifically intended to assess motivations, strategies, and actions in teaching/learning processes. The MESI (STRAT) of Moè et al. (2010) consists of items assessing classroom activities and personalized learning. The inventory provides a detailed inventory of practices used in all aspects of a “lecture” course (this means that it is not suitable for use in courses that are primarily laboratory, seminar, or project courses). Before administering the tools, focus and discussion groups were conducted with teachers and principals to adapt the survey to the target context. Finally, principal component analysis was conducted to reduce the number of practices to a set of more manageable size. The results of the analysis suggested that the items can be fruitfully grouped into three separate domains: co-teaching (examples of such items were “Participate in the didactic-subject group meetings,” “Teach together with another colleague,” “Observe the lessons of the other teachers,” and “Organize activities for students in collaboration with colleagues”), differentiation (e.g., “Use observation to identify students’ needs,” “Define different skills to be achieved depending on the needs of the students,” “Prepare different materials to allow each student to work and learn at his/her personal pace,” and “Positively reinforce students’ commitment”), and classroom management (i.e., “Before explaining a new topic, refer to and discuss topics previously discussed with students,” “Before starting a new topic, understand with the students what the objectives I want to achieve are,” “Organize group work in classroom,” “Provide summary or in-depth materials, such as summaries, work plans, etc.”).

#### Work Engagement, Job Satisfaction and Autonomy

To measure teachers’ psychological working engagement, the present study adopted the Utrecht Work Engagement Scale (UWES-9, Simbula et al., 2011). The nine items of the UWES are grouped into the following three subscales composed of three items in each dimension that reflect the underlying dimensions of engagement: vigor, dedication, and absorption. All items are scored on a 5-point frequency rating scale ranging from 1 (never) to 5 (always). The reliability of the score was higher than standard recommendations (α = 0.84).

Job satisfaction was evaluated with the Teacher Job Satisfaction Scale (TJSS-9, Pepe et al., 2017). The TJSS is a questionnaire aimed at measuring job satisfaction that has been specially developed for use in educational contexts. The TJSS-9 is composed of nine items grouped in three dimensions and is already translated into the target language (e.g., Italian), with three items in each dimension: satisfaction with coworkers, satisfaction with parents, and satisfaction with students’ behaviors. The reliability of the scale was over the recommended threshold (α = 0.75).

The perceptions about the levels of autonomy in teaching were investigated with the Teacher Autonomy Scale (Ulas and Aksu, 2015), which identifies three areas of autonomy in teaching: autonomy in instructional planning and implementation (two items), autonomy in professional development (one item), and autonomy in determining the framework of the curriculum (two items). All items are scored on a 5-point rating scale ranging from 1 (I totally disagree) to 5 (I totally agree). The reliability of the scale was appropriate (α = 0.84).

Finally, the perceptions about social support were investigated by five *ad hoc* items of social support in three different domains: support from colleagues (one item), support from school management (three items), and support from parents (one item). All items were scored on a 5-point rating scale ranging from 1 (I totally disagree) to 5 (I totally agree). The reliability of the scale was in line with recommendations (α = 0.81).

### Structural Equation Modeling

Structural equation modeling (SEM) is a multivariate statistical technique that allows the simultaneous modeling of the relationship between multiple latent and empirical constructs (Byrne, 2016). Thus, SEM is usually selected for simultaneously estimating patterns of relationships between variables under study. SEM is a particular type of path analysis that produces measurement estimation with a better validity and reliability (Hair et al., 2017). In addition, it can also be used to decompose total effects in both direct and indirect effects (Kline, 2015). SEM is fundamentally a hypothesis testing method (i.e., a confirmatory approach) rather than an exploratory approach (e.g., regression analyses). In addition, the SEM technique can explicitly estimate measurement error rather than ignore this issue, as is done with other traditional techniques (Byrne, 2016; Hair et al., 2016).

The statistical significance of the model (i.e., the degree of fit between the conceptual and the empirical model) was evaluated using the following goodness-of-fit indices: root mean square error of approximation (RMSEA, RMSEA < 0.05; Hu and Bentler, 1999); standardized root mean square residual (SRMR, SRMR < 0.05) (Marsh et al., 2004); normed fit index (NFI, NFI > 0.95) (Morin et al., 2013); Tucker–Lewis index (TLI, TLI > 0.95) (Morin et al., 2013); and the comparative fit index (CFI, CFI > 0.95) (Morin et al., 2013). Mahalanobis’s distance (*p* < 0.001) was computed to identify multivariate outliers. No extreme values were removed from the dataset. Next, the distribution of the scores was assessed to evaluate whether they were normally distributed. Given that none of the variables under study displayed kurtosis or skewness values exceeding the recommended limits [−1,+1], the maximum-likelihood method (Gath and Hayes, 2006) was adopted to estimate the parameters for the SEM analysis. In line with indications from the literature (e.g., MacKinnon et al., 2004), 95% confidence limit intervals were computed using both Monte Carlo simulation and bootstrapping methods with a set of random samples (*k* = 500), meaning that the indirect effects for each of the *k* samples and the mean value for the selected pool of samples were analyzed. The software used for all analyses was Amos 23.0 (Arbuckle, 2014).

## Results

Descriptive statistics and zero-order correlations are described in Table 1.

**TABLE 1:** Descriptive statistics and zero-order correlations.

		**1**	**2**	**3**	**4**	**5**	**6**	**7**	**8**	**9**	**10**	**Mean**	**Standard deviation**	**Skewness**
1.	Teachers satisfaction	–										34.2	4.58	–0.418
2.	Social support	0.287^∗∗^	–									18.8	3.84	–0.459
3.	Teachers’ autonomy	0.176^∗∗^	0.195^∗∗^	–								13.3	4.16	0.179
4.	Vigor	0.370^∗∗^	0.316^∗∗^	0.120^∗∗^	–							12.07	2.2	–0.835
5.	Dedication	0.377^∗∗^	0.320^∗∗^	0.147^∗∗^	0.715^∗∗^	–						13.2	1.79	–0.052
6.	Absorption	0.250^∗∗^	0.245^∗∗^	0.116^∗∗^	0.464^∗∗^	0.545^∗∗^	–					12.5	1.93	–0.607
7.	Co-teaching	0.236^∗∗^	0.103^∗∗^	0.011	0.132^∗∗^	0.135^∗∗^	0.145^∗∗^	–				30.2	6.38	0.143
8.	Differentiation	0.276^∗∗^	0.181^∗∗^	0.064	0.240^∗∗^	0.296^∗∗^	0.220^∗∗^	0.355^∗∗^	–			29.3	3.44	–0.228
9.	Classroom management	0.141^∗∗^	0.144^∗∗^	0.134^∗∗^	0.139^∗∗^	0.186^∗∗^	0.139^∗∗^	0.330^∗∗^	0.621^∗∗^	–		62.7	7.4	0.274
10.	Teaching years	0.007	0.011	–0.015	–0.047	–0.124^∗∗^	–0.073	0.082^∗^	0.083^∗^	0.086^∗^	–	15.1	10.72	0.934

*^∗^*P* < 0.05 and ^∗∗^*P* < 0.01.*

From the correlational point of view, zero-order correlations were robust and in line with the expected theoretical directions. The analysis revealed interesting and high associations among work engagement subscales, autonomy, job satisfaction, and perceived social support. Specifically, higher correlations were found between job satisfaction and vigor (*r* = 0.370) and job satisfaction and dedication (*r* = 0.377). We also observed an expected positive correlation between teachers’ autonomy and job satisfaction (*r* = 0.176), as well as between teachers’ autonomy and social support (*r* = 0.195). Finally, the high correlations among the subscales of teaching practices (i.e., between differentiation and classroom management practices, *r* = 0.621) seem to indicate the intrinsic consistency of the teaching practices dimension.

**FIGURE 2:**
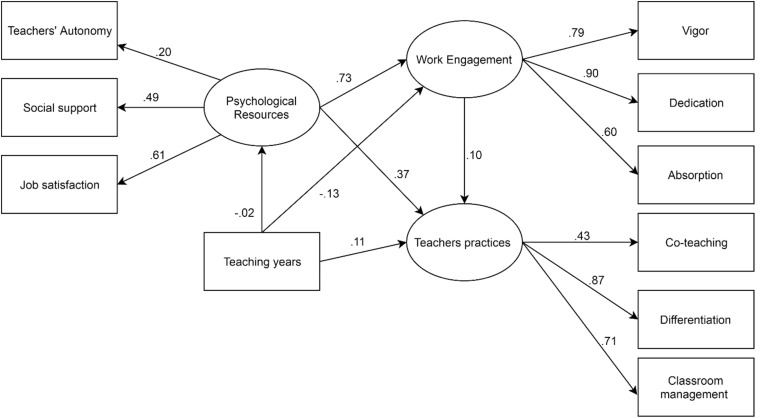
Results of the structural model. Standardized direct effects were reported. Ellipses represented endogenous latent variables. No residual error covariances were fixed.

The tested structural equation model is shown in Figure 2.

From the statistical point of view, regarding the evaluation of the goodness-of-fit indices (relative and absolute), the model was fully supported. The model shows an excellent overlap with all the indicators (relative and absolute): χ^2^(29) = 24.8978.2, *p* < 0.05; NC = 2.7; RMSEA = 0.051, 95% CI = 0.037–0.044, NFI = 0.951, NNFI = 0.950, CFI = 0.968.

Starting from left to right, the total effect of the covariate variable (e.g., teaching years) revealed a small negative total effect on work engagement [β = −0.13, *p* = 0.010; 95% CI (−0.31 to −0.008)] and a positive small total effect on teaching practices [β = 0.11, *p* = 0.014; 95% CI (0.009–0.011)]. This means that the more experienced teachers tend to be less engaged in their work and to use more teaching practices. In contrast, a not statistically significant total effect was found in relation to the appraisal of psychological resources linked to the working environment [β = −0.02, *p* = 0.830; 95% CI (−0.008–0.006)].

With regard to the first hypothesis (H1), the model supported the idea that the appraisal of psychological resources linked to the working environment was directly associated with work engagement. In fact, the total direct effect was positive, large in size, and statistically significant [β = 0.73, *p* = 0.009; 95% CI (1.01–3.25)]. In particular, the analysis suggests that the higher the teachers’ satisfaction, support, and autonomy are, the higher their scores on work engagement. With regard to the second hypothesis (H2), the direct effect of work engagement on teachers’ practices was positive and small but not statistically significant [β = 0.10, *p* = 0.580; 95% CI (−0.444–0.375)], thus not providing support for H2. Finally, the analysis of the total effect of the appraisal of “psychological resources linked to the working environment” on teacher practice was positive, medium in size, and statistically significant [β = 0.45, *p* = 0.005; 95% CI (0.776–3.94)]. The decomposition of the total effect revealed that the working conditions were both directly [β = 0.37, *p* = 0.005; 95% CI (0.418–4.39)] and indirectly [β = 0.08, *p* = 0.032; 95% CI (0.575–0.925)] associated with the repertoire of practices. From this point of view, the analysis suggests that when teachers perceive their work environment as being supportive and satisfying (in the sense of the fulfillment of basic intrinsic psychological needs), they tend to be more engaged and use a larger array of teacher practices, in particular, differentiation [indirect effect, β = 0.39, *p* = 0.014; 95% CI (1.48–7.22)] and classroom management [indirect effect, β = 0.31, *p* = 0.011; 95% CI (0.837–4.14)]. The results provided full support for H3.

## Discussion

The present cross-sectional research investigated the relationships among psychological resources linked to the working environment, work engagement, and classroom practices in a large sample of in-service teachers. In particular, a conceptual model was empirically tested to understand whether and to what extent the way in which teachers perceive psychological resources in their work environment (in terms of job satisfaction, social support, and autonomy) were associated with their work engagement and in turn with the variety of their teaching practices adopted in classrooms. Within the framework of self-determination theories, the rationale for the study was to understand which factors represent promoting conditions for the adoption of heterogeneous repertories in teaching, since the use of different teaching practices and techniques favors the academic outcomes of children (Rowley et al., 2018; Fix et al., 2019). All in all, our findings demonstrate that the presence of psychological resource favors the adoption of heterogeneous and rich repertories of teaching practices, meaning that when teachers perceive their work environment as supportive, satisfying and having a high degree of autonomy (i.e., from another perspective, when their basic psychological needs were fulfilled), they tend to use a larger array of teaching practices.

Concerning the relationship between the psychological resources and work engagement (H1), our results support the idea that a good appraisal of “psychological resources linked to the working environment” was a promoting factor in work engagement in terms of dedication, absorption, and vigor. These results are in line with current literature demonstrating that job resources (Bakker et al., 2007), social support (Saks and Gruman, 2018), and autonomy (Heyns and Rothmann, 2018), might positively affect levels of engagement. Contrary to our expectation (H2), the direct effect of work engagement on teaching practices was small and not statistically significant. Finally, our results support H3, demonstrating that in our sample, intrinsic basic psychological resources (e.g., satisfaction, autonomy, and social support) were more associated to the promotion of teacher practices than were more other psychological aspects (e.g., work engagement). In particular, the analysis suggested that when teachers perceive their work environment as being supportive and satisfying, they tend to be more engaged and in turn use a larger array of teacher practices with students, in particular, differentiation techniques and classroom management. These results are in line with current self-determination theories (Ryan and Deci, 2000) and highlight the role of intrinsic factors in promoting innovative teacher practices (Klaeijsen et al., 2018). Even if the aims of our study were not focused on the role of demographic variables, the structural model also suggests that the length of teaching experience years is associated with work engagement and teacher practices, with more experienced teachers showing less engagement and more use of teaching practices.

## Conclusion

The results of the study show how the evaluation of the psychological working conditions environment (in terms of satisfaction, social support, and professional autonomy) is crucial in promoting teachers’ decision to adopt different or innovative practices. From the point of view of ecological validity, the importance of the study is twofold: one theoretical and one practical. First, from a conceptual point of view, the data from the present study support the indications posited by the SDT that low-ordered basic intrinsic psychological needs are powerful drivers of human behaviors rather than high-ordered needs (e.g., work engagement). In fact, by linking the appraisal of “psychological working conditions” to directly observable tasks (i.e., frequency of teaching practices) of teachers’ jobs, our analysis supports the direct association between the fulfillment of internal psychological states and teacher occupational behaviors in the educational context. Other attempts have been made (see, for instance, Reeve, 2002) to apply SDT to educational settings; however, such studies have focused on students’ behaviors rather than on teacher practices. From this point of view, the present study represents one of the first attempts to apply SDT to the comprehension of teaching behaviors in terms of teaching techniques. Second, and perhaps more germane, our results are an important indicator in terms of planning both preservice and in-service teacher training and promoting the use of innovative techniques. From this perspective, the study shows that to increase the adoption of innovative practices, it is first necessary to work on the perception of “psychological working conditions,” especially in terms of general satisfaction and perception of social support and autonomy. This means that intervention programs aimed at improving the adoption of large and flexible repertories of teaching practices should include work on teachers’ satisfaction, autonomy, and social support. In organizational terms, this would imply a shift from a microsystemic level (e.g., a teacher in interaction with his/her student in a classroom setting) to a macrosystem level (e.g., a teacher in interaction with the organizational system that interacts with students in a classroom setting), putting emphasis on the need for the individual to be supported, satisfied, and more independent and self-directed.

### Limitations, Suggestion, and Future Research

As in the case of other studies, the current research has some limitations that should be addressed. First, all the data were collected by self-reported measures; thus, the presented results may suffer from the common method bias (MacKenzie and Podsakoff, 2012), meaning that some of the statistical variance can be accounted for by the use of self-reported measures rather than by the structural model. Second, it should be noted that all the participants were from Canton Ticino (Switzerland), and generalization to other populations of teachers should be made with caution. In this regard, the finding of the present study should be read more in terms of transferability to similar contexts rather than generalizability to different educational systems or cultural settings. Third, the study was limited to compulsory schools, and higher level education was not taken into consideration. Further studies are welcome to expand current knowledge about teachers’ practices by replicating the present research in other educational grades. Finally, the research design was cross-sectional; consequently, assumptions about cause–effect relationships should not be made. In this regard, the author agrees with the position of Pearl et al. (2016), who state that in cross-sectional design, the relationships between variables should be considered probabilistic rather than deterministic. Beyond the limitations, the present study supported the idea that practice-oriented research and enquiry should be framed within local contexts. In fact, psychological resources linked to working environments represent a powerful “tool” for protecting teachers from the adverse effects of their profession, especially from social-related aspects of their work environments. Teachers should be trusted, supported, and empowered as professionals who can be agents of change contributing to school development. Teachers should be expected, enabled, and encouraged to collaborate; their competences and capacities, as well as their autonomy and accountability, should be considered not only individually but also collectively as part of professional teams. These aspects also affected the variety of practices used by the teachers in classrooms. Future research on teaching practices should consider exploring other psychological resources (e.g., quality of life, principal leadership, collaborative working) linked to the working environment as perceived by workers.

## Data Availability

The datasets generated for this study are available on request to the corresponding author.

## Ethics Statement

The research was conducted by following American Psychological Association, (2010) ethical guidelines and code of conduct. In accordance to the declaration of Helsinki (1964) ethical guidelines, a written informed consent was obtained from all participants. Ethical review and approval was not required for the study on human participants in accordance with the local legislation and institutional requirements (Ethics Committee/IRB).

## Author Contributions

The author confirms being the sole contributor of this work and has approved it for publication.

## Conflict of Interest Statement

The author declares that the research was conducted in the absence of any commercial or financial relationships that could be construed as a potential conflict of interest.
